# A multi-method study of unlicensed medicine risk assessment: a cross-sectional survey of pharmacy procurement leads and policy analysis across NHS trusts in England

**DOI:** 10.1007/s11096-026-02164-6

**Published:** 2026-05-26

**Authors:** Grant Sugiura, Sam Gregory, Francesca Greco, Daniel Grant, Nilesh Patel

**Affiliations:** 1 School of Pharmacy, University of Reading, Reading, UK; 2 Royal Berkshire NHS Foundation Trust, Reading, UK; 3 Oxford University Hospitals NHS Foundation Trust, Oxford, UK; 4 Institute of Pharmaceutical Science, King’s College London, London, UK

**Keywords:** Drug procurement, Hospital (MeSH), National Health Service (NHS), Pharmacy service, Risk assessment, Unlicensed medicines

## Abstract

**Introduction:**

Medicines which lack a marketing authorisation, referred to as unlicensed medicines, are used across healthcare systems internationally. Their use carries recognised quality and safety risks, yet no published evidence describes how healthcare institutions assess and mitigate these risks at a system level.

**Aim:**

This study aimed to explore the processes and variation in risk assessment for unlicensed medicines across NHS trust hospital pharmacies in England.

**Method:**

A multi-method design was used. An online questionnaire was distributed to pharmacy procurement leads across NHS trusts in England (n = 37). Concurrently, unlicensed medicines policies from 40 NHS trusts were analysed using a structured coding framework developed through iterative refinement. The questionnaire collected data on unlicensed medicines risk assessment volume, scope, and governance; policies were analysed to identify organisational intended practice. Datasets were independently analysed and compared at code level across eighteen shared elements to examine variations between practice and policy.

**Results:**

Approximately three quarters of questionnaire respondents (73%; 27/37) reported completing both quality and clinical risk assessments for all unlicensed medicines procured, and most trusts (92%; 34/37) had an unlicensed medicine policy; however, only 65% (24/37) reported clearly defined governance oversight, and 57% (21/37) cited formal approval of unlicensed medicine use. Practices were heterogeneous: risk assessments frequently focused on product identity (e.g., 86%; 32/37, considered country of manufacture) but rarely examined processes of manufacturing or sterilisation. A minority (16%; 6/40) used a formal risk scoring system, and review cycles were inconsistent, with 41% of policies (16/40) not mandating review periods. Policies typically mandated risk assessment and recording on a register but varied in technical detail and rarely indicated that quality checks were required upon receipt. Comparison of findings revealed probable duplication of effort in risk assessment for commonly procured unlicensed medicines and divergence between policy emphasis and practitioner focus.

**Conclusion:**

NHS trusts routinely risk assess unlicensed medicines yet do so independently, with variation in risk assessment content, limited use of structured scoring, and gaps in post-receipt- verification. A national minimum dataset for risk assessment and a shared repository of completed assessments could reduce duplication, improve consistency, and strengthen patient safety.

**Supplementary Information:**

The online version contains supplementary material available at https://doi.org/10.1007/s11096-026-02164-6.

## Impact statements

- To our knowledge, this is the first study to examine how unlicensed medicine risk assessment is undertaken across healthcare organisations, in an area where published evidence is limited.
- The finding that organisations assess the same unlicensed medicines independently, without shared infrastructure, suggests that national coordination could reduce duplication and make better use of resources.
- The variation described here provides a baseline against which any future framework or shared repository for unlicensed medicine risk assessment can be evaluated.

## Introduction

Medicines supplied without a marketing authorisation in the country where they are used are encountered across healthcare systems worldwide, typically where no authorised alternative exists or where an authorised product does not meet a specific clinical need [1, 2]. The terminology and regulatory definition of these products are not harmonised internationally and vary between jurisdictions [3, 4]. Terms used in different settings include ‘unauthorised medicinal products’ by areas under jurisdiction of the European Medicines Agency (EMA), ‘unapproved therapeutic goods’ in Australia, ‘unapproved drugs’ in the United States of America, and ‘unapproved medicines’ in Japan [5–7]. In the United Kingdom (UK), these products are termed ‘unlicensed medicines’, which do not have a marketing authorisation approved by the Medicines and Healthcare products Regulatory Agency (MHRA) or, for centrally authorised products, the EMA [8]. In this study, we have adopted the terminology of ‘unlicensed medicines’ with a focus on the UK National Health Service (NHS). In NHS hospital practice, unlicensed medicines include two main categories. These are (a) imported medicines, which may or may not be licensed in their country of manufacture but are not licensed in the UK, and (b) medicines manufactured to meet a specific patient or organisational need, whether by an MHRA-licensed specials manufacturer, a manufacturer without such a licence, or by extemporaneous preparation in a pharmacy.

The absence of a marketing authorisation means that unlicensed medicines may not have undergone the same rigorous evaluation for safety, efficacy, and manufacturing quality as licensed medicines. Thus, they may be manufactured to a less rigorously assessed quality than licensed medicines [9]. This is recognised as a source of clinical risk across healthcare systems internationally [4, 9]. Despite this, unlicensed medicines use is not uncommon in clinical practice. A systematic review of use of unlicensed medicines in hospitalised paediatric patients reported rates of unlicensed medicines use ranging from 0.2 to 47.9% [10]. Comparable data in adult inpatient populations is lacking. The benefits of unlicensed medicine use must therefore be carefully weighed up against the potential clinical risks they may pose to patients [11–15]. In a nested case–control study of paediatric inpatients, unlicensed medicines were consistently more likely to be implicated in an adverse drug reaction than authorised medicines [11]. Equivalent adverse drug reaction data in adult inpatient populations are absent from the literature. The absence of assured post-market surveillance infrastructure means that risks may remain unidentified unless actively managed at the point of procurement [16].

Regulatory frameworks place the responsibility for risk mitigation on the healthcare organisations that procure and supply these medicines [4]. In the NHS, this is achieved through undertaking risk assessments of unlicensed medicines, which should evaluate both the pharmaceutical quality and the clinical risk profile of an unlicensed medicine prior to procurement and be reviewed periodically to ensure that safety, efficacy and manufacturing quality remain assured [17]. Unidentified and unmanaged risks associated with unlicensed medicines can potentially lead to catastrophic harm to patients and litigation for healthcare organisations, therefore there is a requirement for NHS organisations to risk assess unlicensed medicines [18, 19] There is also a financial burden associated with unlicensed medicines which arise from small batch manufacture and, for imported products, additional transport and third party supplier fees. The development, approval, and ongoing maintenance of the policies and procedures required to govern their use imposes a substantial but often overlooked cost on healthcare providers, borne disproportionately by hospital pharmacy services [17].

Although the need for effective governance of unlicensed medicines has been recognised, published evidence on how risk assessment is carried out in practice remains limited [9]. Existing literature is restricted to a single-centre description of local processes within a broader medicine introduction framework [20] and to our knowledge no study has examined practice across organisations at system level in any country. This is an important gap in the evidence base because, as mentioned previously, in many jurisdictions responsibility for managing these risks rests with the organisations and professionals who procure and supply such medicines. In England, this is particularly relevant because NHS trusts procure and govern unlicensed medicines locally, without a shared national framework. Significant variation in the content of guidance relating to unlicensed medicines has already been documented across NHS primary and secondary care settings, with inconsistency around risk assessment identified as the most significant area of concern in an Appraisal of Guidelines for Research and Evaluation II (AGREE II) analysis of unlicensed medicines policy documents [4]. As yet, no published studies have explored whether NHS trusts undertake risk assessments on unlicensed medicines, how pharmaceutical quality is evaluated prior to procurement, or the degree to which risk assessment practice is consistent across organisations.

### Aim

The aim of the study was to explore the processes and variation in risk assessment for unlicensed medicines across NHS trust hospital pharmacies in England.

## Method

Pragmatism served as the philosophical foundation guiding the conduct of this study. A multi-method approach was used: an online questionnaire sent to pharmacy procurement leads to characterise current practice around unlicensed medicine risk assessment, and a policy content analysis to describe formal governance and recommended processes (Appendix 1 Online Resource 1). The survey and document analysis were conducted concurrently and independently. Following independent analysis of each dataset, items addressing the same verifiable element were identified through a cross mapping conducted post hoc, which formed the basis for comparison between reported practice and documented policy requirements.

### Survey

The reporting of this study followed the Strengthening the Reporting of Observational studies in Epidemiology (STROBE) guidelines [21]. The STROBE checklist is available in Online Resource 2 (Appendix 4). The study invited the participation of pharmacy procurement leads from NHS acute, specialist, and mental health trusts in England. Pharmacy procurement leads were defined as pharmacy staff with delegated responsibility for medicines procurement, and were chosen because they oversee the purchase of unlicensed medicines and are usually the first to identify quality or supply chain risks. The questionnaire was open to procurement leads across England, although the number of individuals performing this role is not centrally recorded. At the time of data collection, England had approximately 120 acute and specialist hospital trusts and around 40 mental health trusts.

Participants from Scotland, Wales, Northern Ireland, and the private sector were excluded. Respondents without direct responsibility for medicines procurement were excluded to obtain a homogenous respondent population aligned with the procurement-led focus of this study. Questionnaire items captured the volume, content, and governance of unlicensed medicines risk assessments, the use of risk registers and ratings, quality control checks, and risk management strategies. A reference list of commonly procured unlicensed medicines was developed from the formulary of one NHS trust in consultation with a pharmacy procurement lead. Unlicensed medicines were identified by generic name and dosage form rather than specific product, manufacturer, or batch. Demographic data captured included region, professional registration, job title, and years in role.

The questionnaire was drafted by the research team and refined following informal expert review for clarity and relevance, after which formal content validity testing was conducted using Lynn’s method [22]. Six secondary care pharmacy professionals were asked to rate each item for clarity, recall, decision and relevance. The questionnaire demonstrated robust content validity (I-CVI 0.78–1.00; S-CVI/UA 0.85; S-CVI/Ave 0.97), exceeding commonly accepted thresholds [23]. The questionnaire is available in Online Resource 1 (Appendix 3).

Participants were recruited through the NHS Chief Pharmacist Network, regional pharmacy procurement leads and NHS trust Research & Development contacts. Invitation emails included the participant information sheet, and a link to the online questionnaire (Jisc Online Survey). The questionnaire was open from April to June 2022, with reminder emails sent one month and two weeks before closure. Recruitment used cascaded distribution through professional networks rather than a defined sampling frame, so the total number of invitees could not be established. As the study was exploratory and descriptive, formal sample size calculation was not appropriate. The aim was to obtain one response from each NHS trust, completed by the individual with lead responsibility for medicines procurement, and to achieve representation across all 10 NHS pharmacy procurement regions in England. Unintended recipients were asked to forward the questionnaire only to the appropriate lead and to submit a single organisational response. Informed consent was obtained before completion, and responses were anonymised. Because recruitment took place through professional networks, the characteristics of non-responders could not be assessed, and representativeness cannot be assured, although this was partly mitigated by distribution across multiple procurement regions and two reminder emails.

Questionnaire data were analysed descriptively using frequencies and percentages. Missing responses were excluded from the denominator for each item, so percentages are based on the number of respondents answering that question.

### Unlicensed medicines policy document content analysis

A policy content analysis using a structured coding framework was conducted on NHS trust unlicensed medicines guidance documents to explore governance recommendations. The study followed four stages: (i) gather documents for analysis, (ii) read documents to develop a generalised understanding, (iii) code documents, and (iv) analyse results [24]. Reporting of the document analysis was guided by the Checklist for Assessment and Reporting of Document Analysis (CARDA) methodology [25]. The completed CARDA checklist is available in Online Resource 4.

Unlicensed medicines policies and guidelines were collected from NHS acute, specialist and mental health trusts in England between August 2021 and July 2022. Documents or guidelines not focused on unlicensed medicines, or aimed at patients rather than NHS staff, such as patient information leaflets, were excluded from the study. Where available, guidelines were obtained through publicly accessible NHS trust websites. Where unlicensed medicine guidance/policies were not readily accessible, freedom of information requests were sent to randomly selected trusts to ensure a distribution of trusts across pharmacy procurement regions in England. Where no response was received after two follow up attempts, an alternative trust from the same region was approached. Eligible documents were existing organisational governance documents that were complete and represented the most recent version available at the time of collection.

For analysis, a structured coding framework of 74 codes across 10 domains was developed iteratively [24]. Codes were generated from a preliminary reading of a purposive sample of policies, informed by the professional and regulatory literature and the research team’s clinical expertise. Two researchers (GS and SG) independently identified and assigned codes, which were then refined through discussion. Items not relevant to the study objectives were excluded. The full coding guide is provided as supplementary material (Online Resource 1, Appendix 4). Ten documents selected at random were then independently coded by both researchers to assess inter-coder reliability. Agreement was 95.6% (708/740 coding decisions). The remaining 30 documents were then divided between the two coders according to capacity (GS 17, SG 13). Ambiguous coding decisions were discussed during regular meetings and resolved by consensus. The presence or absence of each code was recorded as a binary value and data managed within Microsoft Excel. Descriptive statistics were used to summarise the frequency and proportion of each code across the sample. Policy quality appraisal was outside the study scope, as this has been previously evaluated by Donovan et al. [4].

### Comparison of findings

Following independent analysis of each dataset, findings from the questionnaire and policy analysis were compared at code level. The questionnaire items and policy coding framework had been developed independently, and both were informed by the same regulatory and professional literature. Items were classified as equivalent only where the questionnaire item and policy code referred to the same verifiable element. Eighteen of the 74 policy codes had a directly equivalent questionnaire item and so were eligible for comparison. For each shared code, reported practice from the questionnaire was compared with policy requirements identified through the content analysis to examine areas of variation. Where a domain was addressed by only one dataset, the findings were reported within that component. This approach allowed exploration of the relationship between reported practice and organisational policy.

### Ethics approval

Ethical approval for the survey component was granted by the NHS Health Research Authority (21/HRA/4163) and the University of Reading Ethics Committee (UREC 21/51).

## Results

### Survey results

Thirty seven responses were received across all ten NHS pharmacy procurement regions. This represented an estimated 20% response rate, based on the approximate number of eligible NHS trusts at the time of data collection, because the total number of individuals who received the invitation could not be confirmed. The characteristics of non-respondents could not be assessed. Participants comprised 49% (18/37) pharmacists, 46% (17/37) pharmacy technicians and 5% (2/37) procurement or QA specialists.

**Fig. 1:**
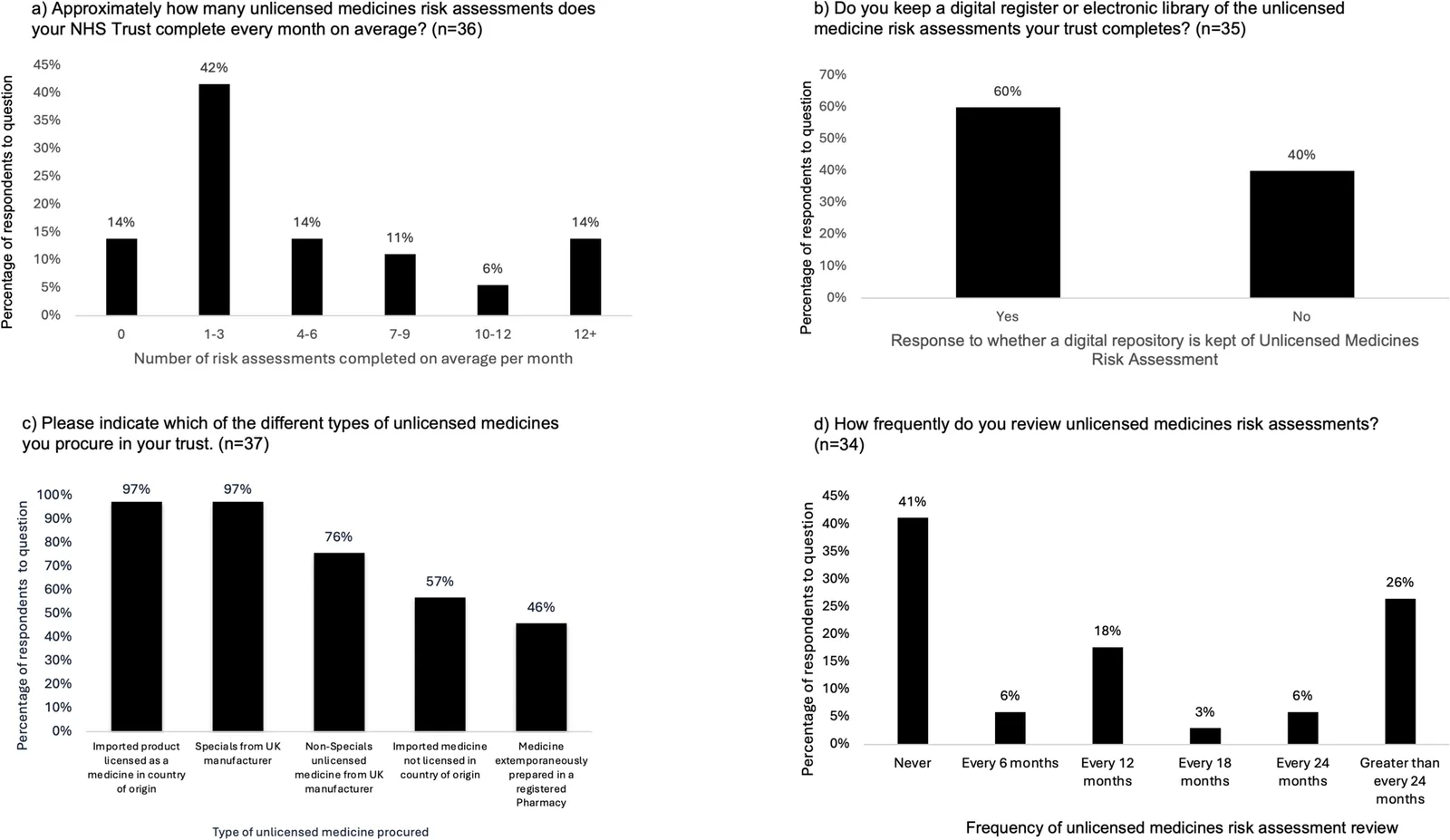
Overview of unlicensed medicines risk assessment activity and oversight across NHS trusts. **a** Volume of risk assessments completed per month; **b** Presence of digital or electronic registers; **c** Types of unlicensed medicines procured; **d** Frequency of risk assessment review. Results illustrate variation in the extent to which NHS trusts undertake, record, and revisit risk assessments for unlicensed medicines

Most participants reported completing 1–3 risk assessments per month, although several participants undertook more than 12 risk assessments per month, indicating substantial variation in workload between organisations (Fig. 1a). Seventy three percent (27/37) of participants stated that they complete quality risk assessments for all unlicensed medicines procured within their trust and the same proportion reported that clinical risk assessments are conducted prior to unlicensed medicine use. Ninety two percent (34/37) of participants stated that their trust has a policy or procedure for unlicensed medicines; however, only 65% (24/37) of participants reported that governance oversight of unlicensed medicine risk assessments was clearly defined in their organisation, and fewer (57%, 21/37) stated that risk assessments were formally approved by a governance group.

The majority of participants indicated that their trust keeps a digital register of completed risk assessments (Fig. 1b). There was both consensus and variation in the types of unlicensed medicines procured by NHS trusts, with almost all respondents reporting sourcing imported products licensed in their country of origin or UK-manufactured “Specials”, but over half also procuring higher-risk products, such as medicines imported without a country of origin licence or medicines extemporaneously prepared in a pharmacy (Fig. 1c). Review arrangements varied considerably, with the largest group of respondents stating that assessments are never reviewed (Fig. 1d).

Unlicensed medicines risk assessments were most commonly completed by pharmacists or pharmacy procurement professionals (62%, 23/37), with pharmacy technicians involved in 35% (13/37) of cases and quality assurance staff in 19% (7/37). Most participants (81%, 30/37) indicated that risk assessments were completed against a standardised proforma, with 58% (21/36) using a formal risk scoring system to categorise risk level numerically (16%, 6/36) or as low/medium/high (33%, 12/36). For unlicensed medicines which are procured, 68% (25/37) of participants stated that quality checks are not completed upon receipt of an unlicensed medicine.

Participants reported procuring a small, overlapping set of high use unlicensed medicines across trusts, including lorazepam injection, metolazone tablets, and fomepizole injection (Table 1). Procurement of products such as pristinamycin tablets and doxycycline injection, was reported by less than a quarter of participants.

**Table 1:** Unlicensed medicines reported by respondents, based on a reference list derived from the formulary of an NHS trust (n = 37)

Unlicensed medicine procured	N (%)
Lorazepam injection	27 (73)
Metolazone tablets	26 (70)
Fomepizole injection	25 (68)
Isoprenaline injection	24 (65)
Aspirin suppositories	23 (62)
Melatonin liquid	23 (62)
Flecainide injection	20 (54)
Mexiletine capsules	20 (54)
Tranexamic acid mouthwash	20 (54)
Artesunate injection	19 (51)
Phentolamine injection	19 (51)
Phytomenadione tablets	19 (51)
Clonazepam injection	18 (49)
Hydrocortisone oral solution	13 (35)
Olanzapine injection	13 (35)
Insulin 50 mL injection	12 (32)
Flucytosine injection	10 (27)
Ibuprofen injection	10 (27)
Idebenone tablets	8 (22)
Vancomycin oral solution	8 (22)
Doxycycline injection	7 (19)
Pristinamycin tablets	6 (16)

Table 2 outlines the factors participants indicated that they include in unlicensed medicines risk assessments. Product identity factors were more commonly assessed than technical aspects of manufacture. Country of manufacture was the most frequently evaluated factor, while supplier licences, transmissible spongiform encephalopathies compliance statements, and the availability of a pharmacopoeial monograph were assessed less frequently. Manufacturing processes and methods of sterilisation were among the least frequently reviewed, suggesting that evaluation of manufacturing quality was relatively limited in scope. Participants also reported using local mitigation measures when risks were identified, most commonly over-labelling products with foreign language packaging, producing local patient information leaflets, and, less often, repackaging products.

**Table 2:** Factors assessed within unlicensed medicines risk assessments reported by pharmacy procurement leads (n = 37)

Factor assessed within risk assessment	N (%)
Country of origin/manufacturing	32 (86)
Manufacturer name	30 (81)
Supplier/importer into the UK name	30 (81)
Shelf life	29 (78)
Storage requirements	29 (78)
Appropriate licences for the activity carried out by the manufacturer	28 (76)
Presence/sample of a product assessment certificate of conformity/analysis	28 (76)
Availability of a summary of product characteristics (or equivalent)	28 (76)
Route of administration	28 (76)
Licensed status in country of origin (such as food supplement, licensed medicine etc.)	27 (73)
Product imagery	25 (68)
Method for reconstitution	23 (62)
Appropriate licences for the activity carried out by the supplier	20 (54)
Presence/sample of a Transmissible Spongiform Encephalopathy (TSE) statement compliance	19 (51)
Availability of a pharmacopeial monograph	17 (46)
Method of sterilisation	10 (27)
Details of method of manufacture	9 (24)
Patient information leaflet	1 (3)

### Document analysis results

Sixteen documents were obtained from trust websites. Freedom of Information requests were submitted to 28 trusts, of which 24 provided eligible documents. The 40 documents analysed had approval dates ranging from 2013 to 2022. Analysis of unlicensed medicine policies (Table 3) revealed substantial variation in content and scope. Definitions and prescriber responsibilities were almost universally included, but the technical and procedural steps required to conduct an unlicensed medicine risk assessment were less represented. Just over half of policies outlined which product or supplier characteristics should be assessed, and only a third provided a standardised risk assessment template. Requirements for post receipt quality checks and allocation of responsibility to a named pharmacy professional were among the least common elements. Although most policies addressed incident reporting and liability, specification of review frequency and documentation requirements varied considerably.

**Table 3:** Policy content analysis of unlicensed medicine policies and procedures across NHS trusts (n = 40). Values represent the number (N) and percentage of policies containing each element

Domain	Content		N (%)
Definitions	Define unlicensed medicine		40 (100)
	Define off-label medicines		37 (93)
	Define specials		33 (83)
	Define extemporaneous preparation		17 (43)
	Define early access medicines scheme		10 (25)
	Define Pre-packs or over labelled packs		15 (38)
Responsibilities	Medical director		15 (38)
	Prescribers		36 (90)
	Drugs and therapeutics committee (or equivalent)		34 (85)
	Chief pharmacist		29 (73)
	Nursing staff		17 (43)
	Pharmacy staff		34 (85)
	Regional quality assurance		4 (10)
Patient consent and information	Patient informed of the intention to use unlicensed medicine		37 (93)
	Patient to participate in making of informed decision/consent		32 (80)
	Documentation in patients’ medical notes		28 (70)
	Restrictions in prescribing		9 (23)
	Information provided on how to access further prescriptions		25 (63)
	Information sharing with GP/Primary care		32 (80)
	Information shared with community pharmacy		5 (13)
	Patient supplied with patient information leaflet		28 (70)
Clinical actions prior to pharmacy purchase of unlicensed medicines	Unlicensed medicine only used if no suitable licensed medicine is available		40 (100)
	Use of unlicensed medicine is approved by drugs and therapeutics committee (or equivalent)		37 (93)
	Declaration of intention to use unlicensed medicine completed by Prescriber		18 (45)
	Use supported by clinical evidence/research/expert body of opinion		36 (90)
	Clinical risk assessment required before use		33 (83)
	Template for clinical risk assessment		24 (60)
	Level of seniority to prescribe unlicensed medicine (consultant, registrar etc.)		19 (48)
Pharmacy actions prior to purchase of unlicensed medicines	Pharmacy quality risk assessment required before use		34 (85)
	Pharmacy quality risk assessment content	Supplier	19 (48)
		Manufacturer	17 (43)
		Country of manufacture	15 (38)
		Certificates of analysis or conformity	15 (38)
		TSE compliance	12 (30)
		Method of sterilisation	1 (3)
		Product specification	15 (38)
		Importer	2 (5)
		Countries of mutual recognition of pharmaceutical quality and manufacturing standards	13 (33)
		Wholesale dealer/MHRA importer licence	9 (23)
		Packaging in English on label	21 (53)
		Translated Patient information leaflet for the specific drug	16 (40)
		Summary of product characteristics	10 (25)
	Flowchart of process provided		22 (55)
	Appropriate licences in place to import or manufacture medicines		19 (48)
	Risk mitigations in place (over labelling/repackaging)		5 (13)
	Placed on an unlicensed medicines risk register		24 (60)
	Allocated a risk rating		26 (65)
	Includes a template risk assessment		13 (33)
	Specifies when unlicensed medicine risk assessment is reviewed		12 (30)
Pharmacy actions upon receipting the product	Quality Control (QC) checks required on receipt of use before issuing to a patient		18 (45)
	Pharmacy professional identified to complete QC checks		15 (38)
	Check that it complies to a product specification		10 (25)
	Labelled in English with English information		15 (38)
	Is quarantined on receipt		19 (48)
	TSE certificate check		6 (15)
	Certificate of Analysis/Certificate of Conformity check on receipt		12 (30)
Incident reporting and liabilities	Reported to medicines information/medicines safety team		12 (30)
	Reported via trust reporting scheme		22 (55)
	Reported to the MHRA via the yellow card scheme		30 (75)
	Liability and legalities around unlicensed medicine		30 (75)

### Comparison of findings of data from survey and unlicensed medicine policies

**Fig. 2:**
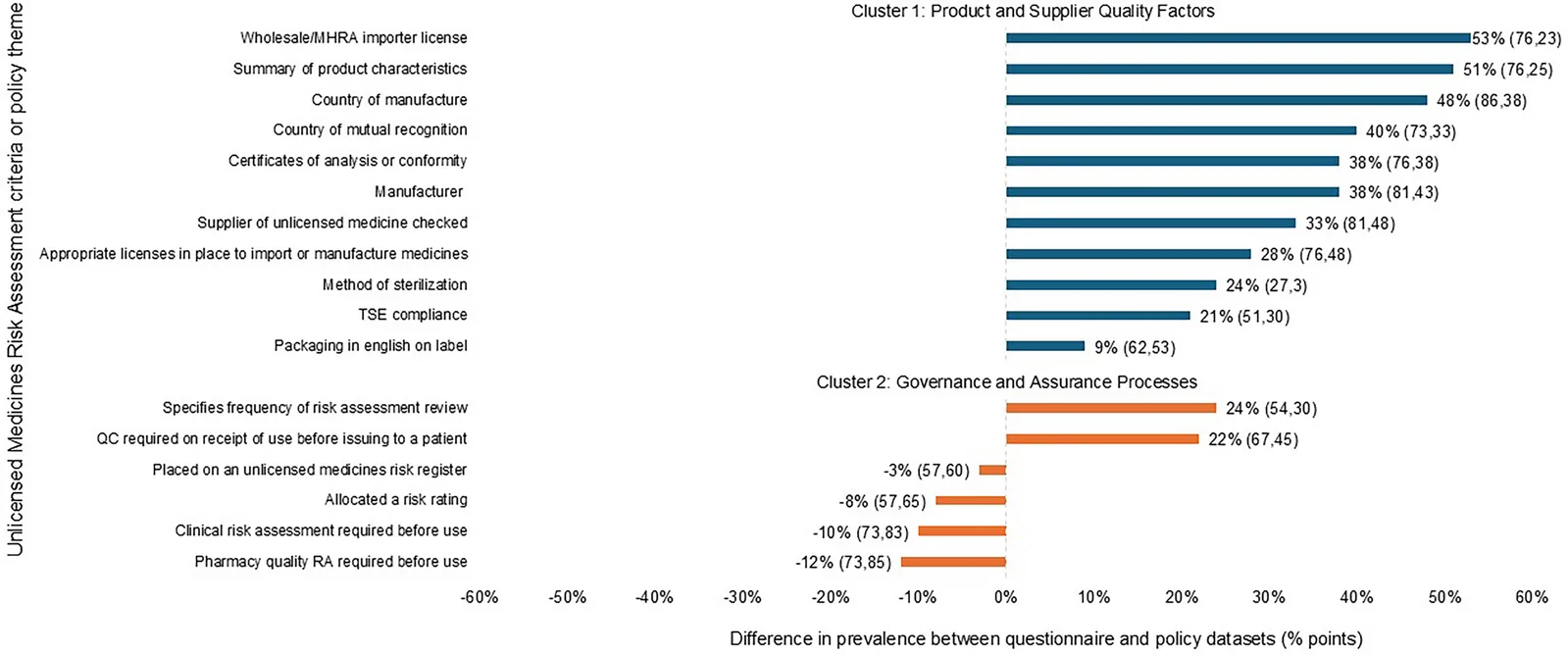
Descriptive comparison of unlicensed medicines risk assessment criteria: survey reported practice (n = 37) versus policy content analysis findings (n = 40). Blue = quality/supplier factors, orange = governance/assurance processes. The first value in the bracket of the data label indicates the percentage within this element for the questionnaire. The second value in the bracket indicates the percentage for this element within the unlicensed medicine policy review

The comparison of the questionnaire findings (n = 37) with the policy analysis (n = 40) enabled a descriptive comparison of the extent to which practice and risk assessment criteria were represented across two independent data sources. Comparisons between proportions should therefore be interpreted as indicating general differences in the prominence of specific criteria across the two datasets, rather than as evidence of direct organisational agreement. Figure 2 illustrates the direction and magnitude of difference for comparable risk assessment criteria.

Requirements for completing formal clinical or quality risk assessments had similar levels of representation across both data sources, with a slightly higher proportion of policies than questionnaire respondents including this requirement. Similar patterns were also observed for assignment of a risk rating and maintenance of an unlicensed medicines risk register. Together, these findings suggest that these procedural features are consistently reflected in both reported practice and written governance documentation.

A different pattern was seen for product and supply chain assurance criteria. Consideration of country of manufacture, supplier and manufacturer identity, and review of certificates of analysis were reported more frequently in questionnaire responses than identified in policies. This suggests that written governance documents may not fully capture the range of technical and supply related factors considered in practice. Scheduled review of risk assessments were reported more often in the questionnaire than specified in policies, suggesting that periodic review may be more established in operational practice than in formal governance documentation.

## Discussion

This study explored the processes and variation in risk assessment for unlicensed medicines across NHS trust hospital pharmacies in England. Using questionnaire data from pharmacy procurement leads alongside a content analysis of organisational policies, variation was found in the content of risk assessments and the governance arrangements that support them. Organisations differed in the factors assessed, how assessments were documented, and the oversight mechanisms in place. Reported practice also diverged from written policy, and the overlap in products, formulations, and suppliers across responding organisations suggests possible duplication of effort at system level.

Where duplication of effort is likely is when the same medicines are being assessed in different organisations without a shared mechanism for recording or sharing those assessments. This may consume pharmacy resource without clear additional safety benefit and echoes the wider concern about unwarranted variation and inefficiency described in NHS systems by the Carter Review [26]. A similar pattern has been described in UK unlicensed medicines guidance. In an analysis of 52 guidance documents, Donovan et al. concluded that healthcare organisations would benefit from agreed core content to improve consistency [4]. The present findings suggest that variation extends beyond written guidance into operational practice.

This variation matters because several of the gaps identified in this study relate directly to product quality assurance. Only around one third of questionnaire participants and half of policies required post-receipt quality checks of unlicensed medicines, and assessments of manufacturing standards or sterilisation processes were among the least frequently reported activities (Table 2). Several participants also reported difficulty accessing manufacturer or pharmaceutical quality information, which may further limit the verification of product quality on receipt. These findings suggest that in a substantial proportion of organisations, unlicensed medicines may reach patients without verification of product quality. For imported products not licensed in their country of origin, or pharmacy prepared products not subject to formal licensing standards, the absence of such checks represents a gap in the safety assurance process. Although this study did not measure adverse drug reactions, unlicensed medicines are known to carry additional quality and safety risks, which makes consistent risk assessment particularly important [3, 11, 14].

Alongside variation in unlicensed medicine risk assessment content, this study also identified weaknesses in governance and review arrangements. Of those participants who reported completing risk assessments, 41% (14/34) stated that they are never reviewed, and only 30% (12/40) of policies specified a review frequency for unlicensed medicines. Inconsistent formal approval processes were also identified across both datasets. These differences may reflect differing organisational risk appetites as well as capacity constraints, as conducting and reviewing risk assessments is considered resource intensive, particularly during periods of increased workload pressures [20, 27].

These findings sit within a wider regulatory and organisational context. Regulatory frameworks in the UK, European Union, Australia, United States, and Japan all permit the supply of medicines outside marketing authorisation under defined conditions, but none prescribe a standardised method for how the procuring organisation should assess pharmaceutical quality or clinical suitability at the point of procurement or use. Responsibility for that activity is therefore left to local determination. In the NHS, the absence of detailed national direction from the MHRA has left hospitals to develop local procedures that vary in scope and depth, echoing earlier concerns that governance processes have not kept pace with the growing complexity of unlicensed medicine use [9]. Similar concerns have also been raised elsewhere. In Japan, the absence of a formal regulatory framework for monitoring unapproved medicines has been linked to difficulties in assuring quality and collecting safety information [28]. In a survey of drug shortage management across 28 countries in Europe and West Asia, Bochenek et al. outlined the need for agreed definitions and standardised methodologies to reduce duplication of effort [29]. A Council of Europe Resolution sought to harmonise quality and safety standards for medicines across member states, but a later survey found heterogenous and incomplete implementation, with variation persisting in areas such as product requirements, and surveillance arrangements [30]. Together, these studies suggest that where no shared framework exists, local approaches tend to develop in different ways, and that issuing guidance alone is unlikely to be sufficient without implementation support, monitoring, and review. Although this study was conducted within the NHS, the governance challenge of organisations independently assessing shared products without common standards or infrastructure is not specific to any one healthcare system, and variation is likely to arise wherever medicines are used without a marketing authorisation and under similar regulatory provisions.

The findings from this study suggest two practical strategies to minimise variation and duplication: (a) shared repositories of risk assessments to allow organisations to use and review existing assessments for the same or similar product, and (b) national or system level guidance setting out a minimum dataset and a standard approach to unlicensed medicine risk assessment. This should build on existing services and platforms for sharing medicines information and professional advice. Any such approach would need to reflect local legal and organisational arrangements, and guidance alone is unlikely to be sufficient without implementation support, monitoring, and review. A further option would be regulatory reform to define minimum standards for risk assessment content and governance, although this would depend on the legal remit of the relevant authority.

This study has some limitations. It was conducted within the organisational and regulatory context of NHS England, so the findings may not transfer directly to settings with different governance structures. Self-selection, the low questionnaire response rate, and reliance on self-reported data may have introduced selection and non-response bias, meaning the findings may either overestimate or underestimate the quality and consistency of unlicensed medicine risk assessment practice. We did not apply a specific theoretical model that might have otherwise informed our approach to study design. The questionnaire sample was limited to pharmacy staff with procurement responsibilities, so the perspectives of other staff involved in the process may be underrepresented. The study also focused on governance within acute hospital trusts and did not examine the wider patient pathway across care settings. The policy sample, derived from Freedom of Information requests and online searches, may have omitted local pharmacy-specific procedures, and the survey and policy datasets were not fully aligned. Finally, duplication was inferred from overlap in reported medicines and suppliers rather than direct comparison of risk assessment records, so it should be interpreted as indicative rather than a precise measure of duplicated effort.

## Conclusion

This multi-method study is, to our knowledge, the first to examine unlicensed medicine risk assessment practice across NHS trusts. The findings of which may also be applicable to settings outside of England. It found variation in the content, depth, and governance of assessments, alongside limited evaluation of pharmaceutical quality before procurement, and a pattern suggestive of duplicated effort. There is no shared national approach. Taken together, these findings suggest that this may create both quality risks and unnecessary demands on local resources. The development and evaluation of minimum standards, alongside systems for sharing assessments across organisations, may help reduce unwarranted variation and support safer practice.

## Supplementary Information

Below is the link to the electronic supplementary material.

Supplementary file1 (DOCX 351 KB)

Supplementary file2 (DOCX 54 KB)

## Data Availability

No datasets were generated or analysed during the current study.
