# Supplementary material for: A multi-method study of unlicensed medicine risk assessment: a cross-sectional survey of pharmacy procurement leads and policy analysis across NHS trusts in England
Source: Int J Clin Pharm. 2026 May 26;48(5):1915–25. doi: 10.1007/s11096-026-02164-6 (PMC13569577; doi:10.1007/s11096-026-02164-6)
Supplement: Supplementary file 1 [file 11096_2026_2164_MOESM1_ESM.docx]

**A multi-method study of unlicensed medicine risk assessment: a cross-sectional survey of pharmacy procurement specialists and policy analysis across NHS trusts in England**

Grant Sugiura^1,2^, Sam Gregory^3^, Francesca Greco^1^, Daniel Grant^1^, Nilesh Patel^4*^

1 School of Pharmacy, University of Reading, Reading, United Kingdom

2 Royal Berkshire NHS Foundation Trust, Reading, United Kingdom

3 Oxford University Hospitals NHS Foundation Trust, Oxford, United Kingdom

4 Institute of Pharmaceutical Science, Kings College London, London, United Kingdom

* author(s) to whom the correspondence should be addressed:

nilesh.patel@kcl.ac.uk

**Keywords**

- Unlicensed Medicines
- Risk Assessment
- Pharmacy Service, Hospital (MeSH)
- Drug Procurement
- National Health Service (NHS)

*Appendix 1. Study design and data sources. Schematic of the multi-method design showing dual data collection (national survey of pharmacy procurement specialists and content analysis hospital unlicensed medicines policy documents) and subsequent comparison of findings to determine convergence and divergence between reported practice and written governance.*

*Appendix 2. Geographic distribution of data sources by NHS pharmacy procurement region. Number of survey respondents and number of trust policy documents included in the content analysis for each pharmacy procurement region in England.*

*Appendix 3. Unlicensed Medicines Risk Assessments Survey*

# Unlicensed Medicines Risk Assessments in NHS Trusts Survey

Most medicines used in the National Health Service (NHS) are licensed medicines, meaning that they have gone through a rigorous process to determine safety, efficacy and quality and thus are permitted to be used under specific circumstances. However, there are instances where no appropriate licensed medicine is available for use to treat a specific patient. In these situations, a doctor may decide to use a medicine that has not been licensed for use in humans (an unlicensed medicine). The risks associated with using unlicensed medicines have been well documented: increased risk of adverse events, potentially poorer quality formulation and absence of regulated manufacturing and supply standards. Therefore, there is a requirement for NHS organisations to risk assess the need and quality of unlicensed medicines to reduce harm to patients, staff, and the organisation.

Between NHS trusts, a lack of consistency has been demonstrated among unlicensed medicine guidelines and practice. This is a concern as trusts may use the same unlicensed medicines but handle them differently with potentially unwarranted variations in practice.

Clear studies of how NHS trusts appropriately manage the risks associated with unlicensed medicines are absent from the literature. There are currently no published studies regarding whether trusts carry out risk assessments and to what extent they evaluate the product before they purchase and use unlicensed medicines, and the steps taken to control the identified risks. There is therefore a need to study this area of unlicensed medicine use within the NHS to demonstrate the current practice and potential variation across England.

This online questionnaire aims to investigate the identification and management of the risks associated with unlicensed medicines by NHS trusts in England through the unlicensed medicines risk assessment process. During this questionnaire you may wish to refer to your trust’s list of approved medication suppliers and/or your trust’s formulary.

*Throughout this questionnaire, we understand that there may be some difficult to answer questions or grey areas. Please answer all questions to the best of your ability. At the end of this questionnaire you will be asked whether you wish to provide a personal email address for future contact to be involved in a further interview study to explore practice and processes of unlicensed medicines risk assessments in more detail, this is voluntary and you can skip this question if you do not wish to be contacted in the future. All data will be processed in such a manner that any personal data submitted can no longer be attributed to a specific set of responses to ensure confidentiality. No personal or organisation-identifiable information will be published in the results.*

*Thank you for taking the time to read the participant information sheet. We require your consent to take part in this study, please review the following statements and select from the options provided. if you have any questions please contact* [*g.sugiura@pgr.reading.ac.uk*](mailto:g.sugiura@pgr.reading.ac.uk)

## Consent statements

I confirm that I have read and understand the Participant Information Sheet (version xx dated xx) for the above study, I have had the opportunity to consider the information, ask questions and have had these answered satisfactorily.

Yes/no

I understand that my participation is voluntary and that I am free to withdraw at any time without giving any reason.
Yes/No

I understand that the study team will use the answers I give for analysis and publication Yes/No

If I have any questions or problems with the questionnaire, I understand that I can contact Grant Sugiura, email: g.sugiura@pgr.reading.ac.uk Yes/No

I understand that it is not possible to withdraw responses after they have been submitted. (However, If a personal email address is provided for a future interview based study, then we will be able to identify your response before analysis and withdraw your response if you wish) Yes/no

If no to the above
Thank you for taking the time to read the Participant Information sheet. Unfortunately, we require your consent to take part in this study. If you have any questions, please contact Grant Sugiura, email: [g.sugiura@pgr.reading.ac.uk](mailto:g.sugiura@pgr.reading.ac.uk)

[OPTIONAL]

I consent to being contacted by the study team for future research (please provide us with an email address and name to contact you on in the future. Please note that providing an email address is not a commitment to participate and you can decline/withdraw as desired). YES/NO

## Unlicensed Medicines Risk Assessments Volume

1) Does your NHS Trust have an unlicensed medicines policy for risk assessing medicines?

- Yes
- No

2) Please indicate below which of the different types of unlicensed medicines you procure in your trust [selection unlimited]

- Imported product licensed as a medicine in country of origin
- Imported medicine not licensed in country of origin
- UK product produced by a manufacturer not under an MHRA manufacturer specials license (e.g. food supplement)
- UK product produced by a manufacturer under an MHRA manufacturer specials license (e.g. a special)
- Medicine extemporaneously prepared in a registered Pharmacy

3) Which of the following suppliers do you use to procure unlicensed medicines? [SELECTION]

- Clinigen
- Mawdsleys
- Durbin
- Alliance Healthcare
- Direct from other NHS trusts
- Direct from Specials manufacturers
- Other (freetext)

4) Below is a list of unlicensed medicines. Please select which of the following you procure into your NHS trust on a regular basis. [SELECTION UNLIMITED]

- Artesunate injection
- Aspirin suppositories
- Clonazepam injection
- Doxycycline injection
- Flecainide injection
- Fomepizole injection
- Flucytosine injection
- Hydrocortisone oral solution
- Ibuprofen injection
- Idebenone tablets
- Human Insulin 50 units in 50mL injection
- Isoprenaline injection
- Lorazepam injection
- Melatonin Liquid
- Metolazone tablets
- Mexiletine capsules
- Olanzapine injection
- Phentolamine injection
- Phytomenadione tablets
- Pristinamycin tablets
- Tranexamic acid mouthwash
- Vancomycin oral solution

5) As an estimate, approximately how many unlicensed medicines risk assessments does your NHS Trust complete every month on average?

[Ordinal scale - 0, 1-3, 4-6, 7-9, 10-12, 12+]

6) What proportion of the unlicensed medicines you procure into your trust **are** risk assessed for **quality** before they are purchased in your trust?

[Percentage brackets 0-20, 21-40, 41-60, 61-80, 81-100]

7) If you do not complete **quality** risk assessments for 100% of all unlicensed medication procured into your hospital, how do you determine which unlicensed medicines are risk assessed and which medications are not risk assessed? [FREETEXT]
8) If **quality** of unlicensed medicines is not risk assessed, do you provide any assurance or checks on unlicensed medicines in another way before they are purchased in your trust?

- Yes
- No

8a) If yes how do you provide this assurance? [FREETEXT}

9) If **quality** of unlicensed medicines procured into your trust is risk assessed, who completes these risk assessments? (select all that apply) [SELECTION – MULTIPLE]

- Pharmacists
- Pharmacy Technicians
- Doctors
- Nurses
- Committee (e.g. drugs and therapeutics committee or equivalent)
- Other (freetext)

## Completion and Content of unlicensed medicines Risk Assessments

10) Does your NHS trust have a standardised proforma for completing unlicensed medicines risk assessments?

- Yes
- No

11) What factors do you review when completing a **quality** risk assessment of an unlicensed medication within your trust. Please select from the list below all the factors which you review [SELECTION MULTIPLE]

- Manufacturer name
- Appropriate licenses for the activity carried out by the manufacturer
- Country of origin/manufacturing
- Licensed status in country of origin (such as food supplement, licensed medicine etc.)
- Supplier/importer into the UK name
- Appropriate licenses for the activity carried out by the supplier?
- Details of method of manufacture
- Method of sterilisation
- Presence/sample of a Transmissible Spongiform Encephalopathy (TSE) statement of compliance
- Presence /sample of a product assessment certificate of conformity/analysis
- Availability of a summary of product characteristics (or equivalent)
- Availability of a pharmacopeial monograph
- Route of administration
- Method for reconstitution
- Shelf life
- Storage requirements
- Product imagery
- Other [Freetext]

12) What sources of information do you access to complete **quality** risk assessment of an unlicensed medication? (i.e. where do you get the information required to assess the above factors) [SELECTION MULTIPLE]

- Manufacturer
- Supplier
- Another NHS Trust
- Regional procurement specialist
- Other [Freetext]

13) Please indicate below how readily accessible you believe the information required to complete unlicensed medicines quality risk assessments is, in general. Please indicate this on the scale below 1 being very inaccessible and 5 being very accessible. [Likert Scale 1 to 5]
14) What proportion of unlicensed medicines **are** risk assessed for **clinical appropriateness** before they are procured into your hospital? [Percentage brackets 0-20, 21-40, 41-60, 61-80, 81-100]
15) If you do not complete **clinical** risk assessments for 100% of all unlicensed medication procured into your hospital, how do you determine which unlicensed medicines are risk assessed and which medications are not risk assessed? [FREETEXT]
16) If you do **clinically** risk assess unlicensed medicines, who completes these clinical risk assessments? [SELECTION – MULTIPLE]

- Pharmacists
- Pharmacy Technicians
- Doctors
- Nurses
- Committee (e.g. drugs and therapeutics committee or equivalent)
- Other (freetext)

17) Are there any other aspects of unlicensed medicines which you risk assess before you procure into your hospital?

- Yes
- No

18) If there are other aspects of unlicensed medicines which you risk assess, please list other aspects you assess: [FREETEXT]

## Risk management and reporting

19) As part of the **clinical and/or quality** risk assessment, do you assign risk ratings/scores to unlicensed medicines ? (i.e. low/medium/high risk, or 1-10 rating]? [Yes/No]
20) If yes, which do you use: [Selection]

- Low/medium/high risk
- Traffic light (Red, Amber, Green)
- 1-10
- Other [FREETEXT]

21) Who reviews the risk assessments and approves the purchasing of the unlicensed medicine?

[FREETEXT]

22) Do you keep a digital register or library of the unlicensed medicine risk assessments your trust completes?

- Yes
- No

23) What is the intended frequency of review for unlicensed medicines risk assessments within your trust? [SELECTION]

- Never
- Every 6 months
- Every 12 months
- Every 18 months
- Every 24 months
- Greater than every 24 months

24) Do all unlicensed medicines procured into your hospital undergo Quality Control inspection checks by a trained professional before they are dispensed to a patient? [SELECTION 1]

- Yes
- No

25) Do you implement any of the below risk control strategies for unlicensed medicines prior to use in patients?

- Over labelling of foreign language products
- Repackaging of medications
- Local production of patient information leaflets
- Other [FREETEXT]

## Participant Demographics

26) Are you a GPhC registered pharmacist or technician?

- Pharmacist
- Technician
- Other
  Specify in comments?

27) Please specify your full job title? [Freetext]
28) How long have you been registered as a healthcare professional?

- <3 years
- 3-5 years
- 5-10 years
- 10-20 years
- >20 years

29) What pharmacy procurement region is your trust part of? (Regions taken from the [SPS Guide to Medicines Procurement in the UK)](https://www.sps.nhs.uk/wp-content/uploads/2017/12/Guide-to-medicine-procurement-in-the-UK-updated-September-2018.pdf)

- North West
- North East
- Yorkshire and Humber
- East Midlands
- West Midlands
- East of England
- London
- South East Coast
- South West
- Thames Valley and Wessex

30) Based from the results of this questionnaire we intend to conduct further qualitative research into the process of assessing unlicensed medicines. If you would be willing to potentially contribute to this research, please could you provide us with an email address to contact you on. Please note that providing an email address is not a commitment to participate and you can decline/withdraw as desired. [FREETEXT]

Version 2.1	23/02/2022

*Appendix 4. Coding Framework for Policy Content Analysis*

## Coding Framework

Each code was applied as a binary decision: present (1) if the policy contained content meeting the definition, or absent (0) if it did not.

| **Ref** | **Domain** | **Code** | **Definition** | **Example** |
| --- | --- | --- | --- | --- |
| 1.1 | **Definitions** | Definition of unlicensed medicines | The policy provides a formal definition of what constitutes an unlicensed medicine. | *'An unlicensed medicine is a medicinal product for human use that does not have a Marketing Authorisation granted by the MHRA or EMA for use within the United Kingdom.'* |
| 1.2 |  | Definition of off-label use of medicines | The policy provides a formal definition of off-label use of a licensed medicine. | *'Off-label use refers to the use of a licensed medicine outside the terms of its Marketing Authorisation, for example for a different indication, dose, or route of administration.'* |
| 1.3 |  | Definition of specials | The policy defines 'Specials' as a category of unlicensed medicine. | *'A Special is a medicine manufactured under an MHRA Manufacturing Specials (MS) licence.'* |
| 1.4 |  | Definition of extemporaneous preparations | The policy defines extemporaneous preparation of medicines. | *'Extemporaneous preparations are medicines prepared or assembled within a pharmacy.'* |
| 1.5 |  | Definition of Early Access to Medicines Scheme | The policy defines or references the EAMS. | *'The Early Access to Medicines Scheme (EAMS) is an MHRA initiative that provides access to medicines that do not yet have a Marketing Authorisation for patients with life-threatening or seriously debilitating conditions.'* |
| 1.6 |  | Definition of pre-packs or TTO packs | The policy defines pre-packs, TTO packs, or over-labelled products in the context of ULMs. | *'Pre-packs or over-labelled packs refer to unlicensed medicines that have been re-labelled with English-language labels to facilitate safe dispensing and supply to patients.'* |
| 2.1 | **Roles and Responsibilities** | Medical / Clinical Director | Responsibilities are defined for the Medical or Clinical Director. | *'The Medical Director is responsible for ensuring that ….* |
| 2.2 |  | Prescribers | Responsibilities are defined for prescribers. | *'The prescriber is responsible for …..'* |
| 2.3 |  | MMC / MMTC / DTC | Responsibilities are defined for the DTC, Medicines Management Committee, or equivalent governance body. | *'The Drug and Therapeutics Committee is responsible for ….* |
| 2.4 |  | Chief Pharmacist / Deputy | Responsibilities are defined for the Chief Pharmacist or Deputy Chief Pharmacist. | *'The Chief Pharmacist is responsible …..'* |
| 2.5 |  | Nursing staff | Responsibilities are defined for nursing staff. | *'Nursing staff are responsible …..'* |
| 2.6 |  | Pharmacy staff | Responsibilities are defined for pharmacy staff (other than the Chief Pharmacist). | *'Pharmacy procurement staff are responsible for ….’* |
| 2.7 |  | Regional Quality Assurance | Responsibilities are defined for the Regional Quality Assurance (QA) service or specialist. | *'The Regional Quality Assurance Specialist should ….'* |
| 3.1 | **Patient Consent and Information** | Patients are informed of the intention to use unlicensed medicines | The policy states that patients must be informed that they will receive an unlicensed medicine. | *'Patients must be informed that the medicine being prescribed does not hold a UK Marketing Authorisation and the reasons why its use is being recommended.'* |
| 3.2 |  | Patient participates in informed decision-making / consent | The policy references the patient's right to participate in informed decision-making or to provide consent. | *'The patient has the right to participate in making a properly informed decision about their treatment and must give consent before an unlicensed medicine is prescribed.'* |
| 3.3 |  | Clinicians document in patient notes that consent has been obtained for use of unlicensed medicine | The policy requires documentation in the patient's medical record that consent has been obtained or ULM use discussed. | *'The prescriber must document in the patient's medical notes that the unlicensed status of the medicine has been explained and that consent has been obtained.'* |
| 3.4 |  | Clinician aware of any restrictions in prescribing | The policy notes any restrictions or limitations on who may prescribe ULMs or under what conditions. | *'Unlicensed medicines may only be prescribed by consultants or senior registrars unless otherwise agreed by the Drugs and Therapeutics Committee.'* |
| 3.5 |  | Information is given on how to access further prescriptions | The policy provides information about how the patient can access further supplies of the ULM. | *'Patients should be provided with information on how to access further prescriptions, including whether the medicine will be prescribed in primary care or through a hospital outpatient pharmacy.'* |
| 3.6 |  | Information is handed over to primary care on how the drug is used, including monitoring and risks | The policy requires that information about the ULM is shared with the patient's GP or primary care provider. | *'On discharge, the prescriber must ensure that the patient's GP is informed of the unlicensed medicine, including the clinical indication, dose, monitoring requirements, and any associated risks.'* |
| 3.7 |  | Information is shared with community pharmacies | The policy requires that information is shared with the community pharmacist. | *'Where a patient is to receive an ongoing supply of an unlicensed medicine from a community pharmacy, the prescriber or hospital pharmacist should ensure that the community pharmacist is informed of the unlicensed status and any special handling requirements.'* |
| 3.8 |  | Patient is supplied with an unlicensed medicine patient information leaflet | The policy requires that a patient information leaflet is provided to the patient. | *'Patients must be supplied with an appropriate patient information leaflet. Where a manufacturer's PIL is not available in English, a locally produced information leaflet should be provided.'* |
| 4.1 | **Clinical Actions Prior to Pharmacy Purchase** | Assurance that no suitable licensed medicinal product is available to meet the clinical needs of the patient | The policy states that ULMs should only be used where no suitable licensed alternative exists. | *'An unlicensed medicine should only be used where there is no suitable licensed medicine available that meets the clinical needs of the patient.'* |
| 4.2 |  | Use of a new unlicensed product is approved by MMTC / DTC / MI etc. | The policy requires that the use of a ULM is approved by the DTC or equivalent governance body. | *'Use of a new unlicensed medicine must be approved by the Drugs and Therapeutics Committee before it may be prescribed within the Trust.'* |
| 4.3 |  | Declaration forms are completed by the prescriber | The policy requires the prescriber to complete a formal declaration or request form. | *'The prescriber must complete an Unlicensed Medicine Declaration Form prior to first use, detailing the clinical justification, patient details, and intended duration of treatment.'* |
| 4.4 |  | Use is justified by clinical evidence or research | The policy requires clinical justification supported by evidence, research, or expert opinion. | *'The prescriber must ensure that the use of the unlicensed medicine is supported by a sufficient body of clinical evidence.'* |
| 4.5 |  | Clinical risk assessment required before use | The policy requires a clinical risk assessment to be completed before ULM use. | *'A clinical risk assessment must be completed by the prescriber to evaluate the potential risks and benefits of using the unlicensed medicine for the individual patient.'* |
| 4.6 |  | Template included for documenting clinical justification / risk assessment | The policy includes or references a template for documenting clinical justification or clinical risk assessment. | *'A clinical risk assessment form (Appendix 2) must be completed by the prescriber, documenting the clinical indication, evidence base, patient-specific risks, and proposed monitoring plan.'* |
| 4.7 |  | Level of authorisation to prescribe is documented, such as consultant or junior doctor | The policy specifies the minimum level of clinical seniority required to prescribe a ULM. | *'Unlicensed medicines should normally be initiated by a consultant or specialist registrar. Junior medical staff may prescribe continuation supplies with consultant authorisation.'* |
| 5.1 | **Pharmacy Actions Prior to Purchase of ULM** | Risk assessment must be completed | The policy requires a pharmaceutical quality risk assessment to be completed before purchase or use of a ULM. | *'A pharmaceutical quality risk assessment must be completed and approved before any unlicensed medicine is procured for use within the Trust.'* |
| 5.1.1 |  | Unlicensed Medicine Supplier | The risk assessment includes assessment of the supplier. | *'The risk assessment must include an evaluation of the supplier, including their name, licensing status, and track record of supply.'* |
| 5.1.2 |  | Unlicensed medicine Manufacturer | The risk assessment includes assessment of the manufacturer. | *'The name of the manufacturer should be recorded as part of the pharmaceutical quality risk assessment.'* |
| 5.1.3 |  | Country of manufacture | The risk assessment includes consideration of the country of manufacture. | *'The country of manufacture should be identified and considered as part of the risk assessment, with particular attention to regulatory standards in that jurisdiction.'* |
| 5.1.4 |  | Certificates of analysis or conformity | The risk assessment includes review of CoA or CoC. | *'A certificate of analysis or certificate of conformity must be obtained from the supplier and reviewed as part of the risk assessment to confirm that the product meets the agreed specification.'* |
| 5.1.5 |  | TSE compliance | The risk assessment includes a TSE compliance statement. | *‘A TSE compliance statement must be obtained from the manufacturer and included in the risk assessment.'* |
| 5.1.6 |  | Method of sterilisation | The risk assessment includes assessment of the method of sterilisation. | *'For sterile products, the risk assessment should include consideration of the method of sterilisation used and whether this provides adequate sterility assurance.'* |
| 5.1.7 |  | Product specification | The risk assessment includes review of the product specification. | *'A product specification detailing the formulation, strength, presentation, and excipients should be obtained and reviewed as part of the risk assessment.'* |
| 5.1.8 |  | Importer | The risk assessment includes assessment of the importer. | *'Where the product is imported, the identity and licensing status of the importing agent should be assessed as part of the risk assessment.'* |
| 5.1.9 |  | Countries of mutual recognition of pharmaceutical quality and manufacturing standards | The risk assessment considers whether the product is manufactured in a country with mutual recognition of pharmaceutical standards. | *'Consideration should be given to whether the country of manufacture has mutual recognition arrangements with the UK for pharmaceutical quality and manufacturing standards.'* |
| 5.1.10 |  | Wholesale dealer / MHRA importer licence | The risk assessment includes verification of wholesale dealer or MHRA importer licence. | *'The risk assessment should confirm that the supplier holds an appropriate wholesale dealer's licence (WDA) or MHRA manufacturer's/importer's authorisation for the activity being carried out.'* |
| 5.1.11 |  | Packaging in English on label | The risk assessment considers whether the product packaging and labelling are in English. | *'The risk assessment should consider whether the product is labelled in English. Where labelling is in a foreign language, over-labelling or re-labelling strategies should be identified.'* |
| 5.1.12 |  | Translated patient information leaflet for the specific drug | The risk assessment considers the availability of a translated PIL. | *'The risk assessment should consider whether a patient information leaflet is available in English. Where a translated PIL is not available, the pharmacy department should produce a local patient information leaflet.'* |
| 5.1.13 |  | Summary of product characteristics | The risk assessment considers the availability of a Summary of Product Characteristics (SPC) or equivalent. | *'A Summary of Product Characteristics (SPC), or equivalent document from the country of origin, should be obtained and reviewed as part of the pharmaceutical quality risk assessment.'* |
| 5.2 |  | Flowchart provided for the procedure of obtaining an unlicensed medicine | The policy includes a flowchart or process diagram for obtaining a ULM. | *Flowchart present* |
| 5.3 |  | Supplier holds appropriate licence to import or manufacture product | The policy requires verification that appropriate licences are held by the supplier/manufacturer. | *'Before purchasing, pharmacy staff must confirm that the supplier holds all appropriate licences for the manufacture, import, or wholesale distribution of the product.'* |
| 5.4 |  | Strategies identified to reduce risk, such as over-labelling of foreign products, repackaging of products, or production of staff information leaflets | The policy identifies strategies to reduce risk such as over-labelling or repackaging. | *'Where a product is labelled in a foreign language, the pharmacy department should arrange for over-labelling with English-language labels that include at minimum the product name, strength, dose, and expiry date.'* |
| 5.5 |  | Product placed on an unlicensed medicine risk register | The policy requires ULMs to be recorded on a risk register. | *'All unlicensed medicines for which a risk assessment has been completed must be recorded on the Trust's Unlicensed Medicines Risk Register.'* |
| 5.6 |  | Product allocated a risk level, such as numerical or high / medium / low | The policy requires that ULMs are allocated a risk level or rating. | *'Each unlicensed medicine must be assigned a risk rating (low, medium, or high) based on the outcome of the pharmaceutical quality risk assessment. The risk rating will determine the frequency of review and level of ongoing monitoring required.'* |
| 5.7 |  | Unlicensed medicines risk assessment must be approved | The policy specifies who is responsible for approving the pharmaceutical quality risk assessment. | *'Completed pharmaceutical quality risk assessments must be reviewed and approved by…..'* |
| 5.8 |  | Review period for unlicensed medicines risk assessment specified | The policy specifies a review period or trigger for reassessing the ULM risk assessment. | *'Unlicensed medicine risk assessments must be reviewed at least every year, or sooner if there is a change in supplier, manufacturer, or product specification.'* |
| 6.1 | **Quality Control on Receipt** | Quality control checks are required on receipt of the medication | The policy requires QC checks to be performed when the ULM is received. | *'Quality control checks must be completed on receipt of all unlicensed medicines before the product is released for dispensing. Products must be quarantined until checks are satisfactorily completed.'* |
| 6.2 |  | Identified pharmacy professional who will complete quality control checks | The policy identifies who is responsible for completing QC checks. | *'Quality control checks on receipt of unlicensed medicines must be completed by a pharmacist or suitably trained pharmacy technician designated by the Chief Pharmacist.'* |
| 6.3 |  | Product complies with the product specification | The policy requires checking that the received product complies with the agreed specification. | *'On receipt, the product must be checked to ensure that it complies with the product specification documented in the pharmaceutical quality risk assessment, including formulation, strength, and presentation.'* |
| 6.4 |  | Product labelled in English or supplied with English information | The policy requires checking that the product is labelled in English or has English information. | *'On receipt, pharmacy staff should confirm that the product is labelled in English or has been appropriately over-labelled with English-language information.'* |
| 6.5 |  | Product placed into quarantine area if appropriate | The policy requires ULMs to be quarantined on receipt. | *'Unlicensed medicines must be placed in a designated quarantine area on receipt and must not be released for dispensing until all quality control checks have been satisfactorily completed.'* |
| 6.6 |  | TSE certificate obtained | The policy requires a TSE certificate check upon receipt. | *'Where the product contains animal-derived ingredients, the TSE compliance certificate should be verified against the risk assessment documentation on receipt of each delivery.'* |
| 6.7 |  | Certificate of Analysis / Certificate of Conformity obtained | The policy requires CoA or CoC to be checked upon receipt. | *'On receipt, the batch-specific certificate of analysis or conformity must be obtained and checked to confirm that the product meets the specification documented in the risk assessment.'* |
| 7.1 | **Dispensing Records** | Record of the manufacturer / supplier is kept | Dispensing records include the manufacturer or supplier name. | *'The dispensing record must include the name of the manufacturer and/or supplier of each unlicensed medicine dispensed.'* |
| 7.2 |  | Date of issue recorded | Dispensing records include the date of issue. | *'The date of issue must be recorded for each supply of an unlicensed medicine.'* |
| 7.3 |  | Patient details recorded | Dispensing records include patient details. | *'Patient details including name, hospital number, and ward must be recorded for each supply of an unlicensed medicine.'* |
| 7.4 |  | Prescriber's name recorded | Dispensing records include the prescriber's name. | *'The name of the prescriber must be recorded in the unlicensed medicines dispensing log.'* |
| 7.5 |  | Quantity recorded | Dispensing records include the quantity dispensed. | *'The quantity of unlicensed medicine dispensed must be recorded.'* |
| 7.6 |  | Batch recorded | Dispensing records include the batch number. | *'The batch number of the unlicensed medicine dispensed to the patient must be recorded to enable traceability.'* |
| 7.7 |  | Expiry date recorded | Dispensing records include the expiry date. | *'The expiry date of the unlicensed medicine dispensed must be recorded.'* |
| 8.1 | **Ongoing Record Keeping** | Records kept to meet current MHRA guidelines and legislation | Records are kept to meet MHRA guidelines and legislation. | *'All records relating to unlicensed medicines must be maintained in accordance with current MHRA guidelines and relevant legislation.'* |
| 8.2 |  | Records retained for a period of 5 years | Records are retained for a period of 5 years. | *'All records relating to unlicensed medicines must be retained for a minimum period of 5 years.'* |
| 8.3 |  | Records retained for another specified period of time | Records are retained for a specified period other than 5 years. | *'Records must be retained for a minimum of 11 years in accordance with Trust records management policy.'* |
| 9.1 | **Incident Reporting and Liabilities** | Problems with the use of unlicensed medicines are reported to MI or medicines safety | The policy requires problems or incidents with ULMs to be reported to the MI or medicines safety team. | *'Any problems or concerns relating to the use of an unlicensed medicine should be reported to the Medicines Information Service or Medicines Safety Team for investigation and further action.'* |
| 9.2 |  | Incidents relating to adverse reactions for unlicensed medicines are reported via the trust-specific incident reporting scheme | The policy requires incidents to be reported through the Trust's internal incident reporting system. | *'Any adverse reactions or quality defects associated with unlicensed medicines must be reported via the Trust's incident reporting system in accordance with local policy.'* |
| 9.3 |  | Incidents relating to adverse reactions for unlicensed medicines are reported to the MHRA via the Yellow Card Scheme | The policy requires reporting of adverse reactions to the MHRA via the Yellow Card Scheme. | *'All suspected adverse drug reactions associated with unlicensed medicines must be reported to the MHRA via the Yellow Card Scheme.'* |
| 9.4 |  | Liability and legality of actions are covered | The policy addresses legal liability and medico-legal implications of using ULMs. | *'Prescribers should be aware that when prescribing an unlicensed medicine, the prescriber bears greater professional and legal responsibility than when prescribing a licensed medicine within the terms of its marketing authorisation.'* |
| 10.1 | **Training** | Details of training for pharmacy staff | Training requirements are specified for pharmacy staff. | *'Pharmacy staff involved in the procurement, risk assessment, or dispensing of unlicensed medicines must receive appropriate training.'* |
| 10.2 |  | Details of training for medical staff | Training requirements are specified for medical staff. | *'Medical staff who prescribe unlicensed medicines should receive training on the ULM policy, including consent requirements.'* |
| 10.3 |  | Details of training for nursing staff | Training requirements are specified for nursing staff. | *'Nursing staff who administer unlicensed medicines should be made aware of the unlicensed status and specific requirements.'* |
| 10.4 |  | Details of training for other staff groups | Training requirements are specified for other staff groups. | *'All relevant staff groups involved in the handling or administration of unlicensed medicines should receive role-specific training.'* |

Version 3.0	22/04/2022
